# Identification of miRNAs involved in pear fruit development and quality

**DOI:** 10.1186/1471-2164-15-953

**Published:** 2014-11-03

**Authors:** Jun Wu, Defu Wang, Yufeng Liu, Long Wang, Xin Qiao, Shaoling Zhang

**Affiliations:** College of Horticulture, State Key Laboratory of Crop Genetics and Germplasm Enhancement, Nanjing Agricultural University, Nanjing, 210095 China; BGI-Shenzhen, Shenzhen, 518083 China

**Keywords:** Pear, Fruit development, High-throughput sequencing, miRNA, Lignin synthesis, Sugar and acid

## Abstract

**Background:**

MicroRNAs (miRNAs) are a class of small, endogenous RNAs that take part in regulating genes through mediating gene expressions at the post-transcriptional level in plants. Previous studies have reported miRNA identification in various plants ranging from model plants to perennial fruit trees. However, the role of miRNAs in pear (*Pyrus bretschneideri*) fruit development is not clear. Here, we investigated the miRNA profiles of pear fruits from different time stages during development with Illumina HiSeq 2000 platform and bioinformatics analysis. Quantitative real-time PCR was used to validate the expression levels of miRNAs.

**Results:**

Both conserved and species-specific miRNAs in pear have been identified in this study. Total reads, ranging from 19,030,925 to 25,576,773, were obtained from six small RNA libraries constructed for different stages of fruit development after flowering. Comparative profiling showed that an average of 90 miRNAs was expressed with significant differences between various developmental stages. KEGG pathway analysis on 2,216 target genes of 188 known miRNAs and 1,127 target genes of 184 novel miRNAs showed that miRNAs are widely involved in the regulation of fruit development. Among these, a total of eleven miRNAs putatively participate in the pathway of lignin biosynthesis, nine miRNAs were identified to take part in sugar and acid metabolism, and MiR160 was identified to regulate auxin response factor.

**Conclusion:**

Comparative analysis of miRNAomes during pear fruit development is presented, and miRNAs were proved to be widely involved in the regulation of fruit development and formation of fruit quality, for example through lignin synthesis, sugar and acid metabolism, and hormone signaling. Combined with computational analysis and experimental confirmation, the research contributes valuable information for further functional research of microRNA in fruit development for pear and other species.

**Electronic supplementary material:**

The online version of this article (doi:10.1186/1471-2164-15-953) contains supplementary material, which is available to authorized users.

## Background

Since the identification of the first microRNAs (miRNAs) as key regulatory molecules in the pathway controlling the timing of larval development in *Caenorhabditis elegans* [1], great progress has been made in the understanding of miRNA function in various species. Ha and Kim reported the biogenesis of miRNA in animals and pointed out that dis-regulation of miRNAs is associated with many human diseases [2]. In plants, the molecular events underlying miRNA biogenesis and degradation, and miRNA-mediated gene silencing were also reported [3], such as, MiRNAs are involved in plant development, signaling, abiotic stresses and symbiotic relationship regulation [4]. Intensive studies indicate that miRNAs are involved in post-transcriptional regulation and appear to be one of the most significant regulatory mechanisms [1, 5]. Currently, more than 30,424 mature miRNAs have been identified from 206 species including vertebrates, flies, worms, plants and viruses (miRBase release 20, June 2013, http://www.mirbase.org/). High evolutionary conservation among different plant species provides the premise for miRNA identification. In plants, miRNAs are single strand, usually 21–22 nt long small RNA molecules that are processed by Dicer-like 1(DCL1). MiRNA/miRNA* duplexes, which contain the mature miRNA, are processed from pre-miRNA which is produced from primary miRNA [6]. One of the strands of the generated miRNA/miRNA* duplex is incorporated into the RNA-induced silencing complex (RISC). This strand is usually the mature miRNA strand and the miRNA* strand gets degraded [7]. By cleavage of target mRNAs [8], translational repression [9] or transcriptional inhibition [10], mature miRNAs suppress the expression of their target genes. Some plant miRNAs physically clustered together as a polycistron, suggesting that these miRNAs potentially regulate gene expression coordinately [7].

A number of studies have suggested that miRNAs play an important role in regulating development of plants, the response to environment and many other biochemical reactions [11–15]. For example, Gao et al. [16] reported that miR319/miR319a/miR319e may contribute to an increase in imperfect flower ratios in the pistil of Japanese apricot development. MiR172 functions in regulating the transition between developmental stages and in specifying floral organ identity by regulating expression of AP2-domain-containing transcription factors in monocots and dicots [17]. Karlova et al. [18] reported that *CNR* and *SIAP2a,* known as the auxin response factor (ARF) and ripening regulators, were actively modulated by miR156/157 and miR172 during tomato ripening. Moxon et al. [19] validated that a member of the constitutive triple response (CTR) family is involved in tomato fruit ripening, which is the target of the novel miRNA, and a higher expression pattern of miR390 was found in small fruit than in leaves and flowers. Over expressing an AtMIR156b precursor generated abnormal flower and fruit morphology in tomato by targeting *S.lycopersicum* SBP genes [20]. Itaya et al. [21] reported that miR167 is involved in auxin response and APETALA2 (AP2) was the target gene of miR172. In strawberry [8], two members of miR159 (Fa-miR159a and Fa-miR159b) were identified interacting with *Fa-GAMYB* during the course of berry receptacle development and cooperatively changed GA endogenous levels. In sweet orange, Csi-miR164 showed a high expression level in fruit ripening and targeted the NAC transcription factor [22].

Pear (*Pyrus spp*) with more than 3,000 years of cultivation history is regarded as one of the most important fruit crops and cultivated worldwide in 76 countries. Rich in vitamin C, dietary fiber, sugar, protein, and many other nutrients, pear fruit has consistently been consumed fresh, canned, and dried and is believed to benefit human health. Small RNAs have been annotated for pear genome in our previous research study [23], and the genome wide identification of pear miRNAs was recently reported recently [24]. However, the function of miRNAs in pear fruit development remains unknown and specific miRNAs for fruit are expected to be identified. In this study, we investigated the miRNA profiles of pear fruits from different developmental stages with Illumina HiSeq 2000 platform, combined with bioinformatics analysis and experimental verification, and it is the first to prove that miRNAs are widely involved in the regulation of pear fruit development and quality.

## Results and discussion

### Small RNA library preparation and sequencing

Total RNA extracted from pear fruit samples, which were collected at different developmental stages from 15 days after flowering (DAF) to 167 DAF, was employed to build small RNA libraries for sequencing. Total reads, ranging from 19 million to 25 million of the six libraries, were generated from Illumina HiSeq 2000 (Table 1). After removing adaptors, poly A reads and reads of less than 18 nt, clean reads varied from 18 million to 25 million, which occupied on average 97.81% in total reads of the six libraries, were collected. Approximately 3.29% (747,350) of the clean reads could be mapped to known noncoding RNAs (rRNA, snRNA, snoRNA, tRNA) deposited in the NCBI. There were 1,217,957 reads on average identified to be putative known miRNAs, and an average of 8,596,939 identified to be un-annotated miRNAs (Table 1). Using SOAP2 software, more than 15 million clean reads (69.335%) were perfectly mapped to the pear genome [23].

**Table 1:** **Categorization of reads of small RNAs in pear fruit at various developmental stages DAF: days after flowering**

Category	Development stages						Average
	15DAF	36 DAF	81 DAF	110 DAF	145 DAF	167 DAF	
Total reads	22,354,861	23,934,357	19,030,925	25,576,773	23,934,213	24,527,030	23,226,360
Clean reads	21,612,084	23,153,525	18,618,514	25,086,346	23,575,395	24,263,969	22,718,306
Exon_antisense	319,460	308,465	218,280	257,309	215,078	220,482	256,512
Exon_sense	509,150	453,742	341,272	398,473	365,311	350,102	403,008
Intron_antisense	377,104	395,221	298,440	378,094	331,083	360,635	356,763
Intron_sense	797,178	619,168	528,500	614,109	662,064	599,612	636,772
Known miRNAs	1,147,166	1,014,459	1,271,600	1,590,575	1,170,540	1,113,401	1,217,957
rRNA etc.*	1,158,744	756,242	617,356	708,315	607,519	635,925	747,350
Repeat	9,514,359	10,881,244	8,544,263	11,564,973	11,161,868	11,351,321	10,503,005
Un-annotated	7,788,923	8,724,984	6,798,803	9,574,498	9,061,932	9,632,491	8,596,939

*rRNA/snRNA/snoRNA/tRNA considered.

**Figure 1:**
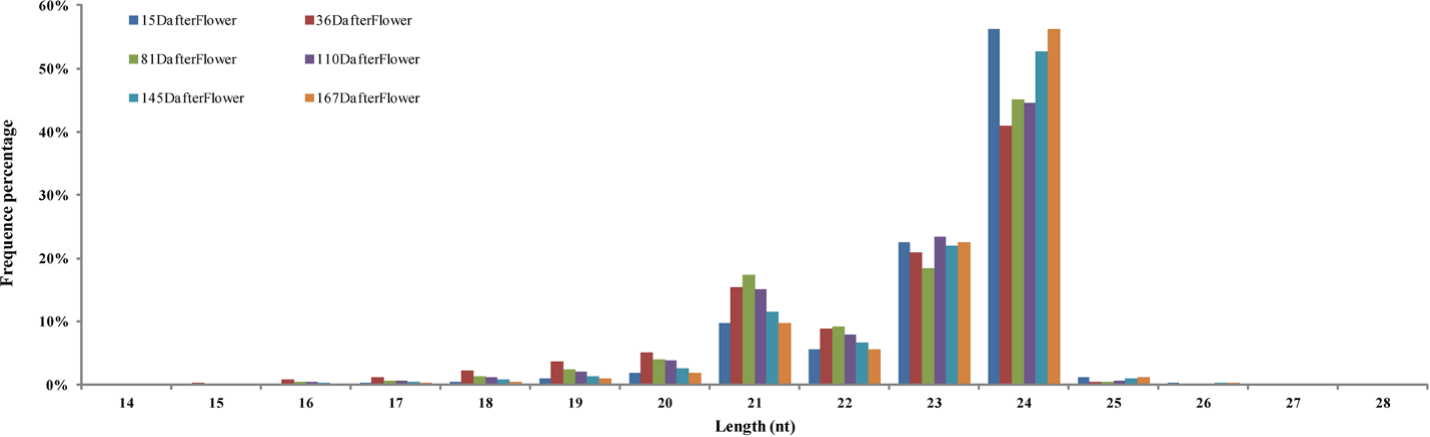
**The length distribution of small RNAs.** Y-axis represents percentages of small RNA identified in the study. X-axis represents the length of small RNA. Six libraries are shown by different colors. The figure shows that 21 nt to 24 nt were highest frequency (nearly 90%) among all of the clean reads in each library, with 24 nt as the most abundant (close to 50%) small RNA length, followed by 23 nt, 21 nt, and 20 nt, in that order.

The number of reads with different lengths of the six small RNA libraries was counted (Figure 1). It was observed that small RNAs with length ranging from 21 nt to 24 nt were most frequent (nearly 90%) of all the clean reads of each library, with 24 nt the most abundant (close to 50%) small RNA length, followed by 23 nt, 21 nt, and 20 nt, in that order. The result was consistent with previous reports in various species, including tomato [19], *Arabidopsis thaliana* [25, 26], *Citrus trifoliate* [27], *Medicago truncatula* [28, 29], *Citrus sativus* [30], and maize [10] which all suggest the most abundant small RNA length is 24 nt.

### Identification of conserved miRNAs in pear fruit

In order to identify the known miRNAs in pear fruit, the clean reads of each library were compared to other plant miRNAs deposited in miRBase 20.0. Following the BLASTn searches and further sequence analysis, on average more than 1 million total reads for six libraries were perfectly matched to 362 known miRNAs (Additional file 1). Among these, 60 miRNAs were identified as 55 known miRNA families deposited in miRBase. The numbers of miRNA in one miRNA family were various. As listed in Additional file 1, five families, including miR156, miR164, miR167, miR2111 and miR4414, have two miRNAs each. The rest of the 50 families have one miRNA each, and there were 302 conserved miRNAs with no match to any family, possibly due to the variation between different species.

The location of 362 conserved miRNA was shown according to the pear genome data (Additional file 1). From these, 266 miRNAs were located on chromosomes or scaffolds, and multiple transcript sites for all 266 miRNAs ensured the miRNA gene expression and important regulation function. However, this result showed great difference with rice, in which 69% of mapped small RNAs have a unique genome location [9]. Whether the multiple or unique transcripts of miRNA have a special function is still unknown. The remaining 96 pear fruit miRNAs did not map to chromosome sites due to incomplete genome annotation. Except for the 96 miRNAs with unknown transcript sites, miR4250 had the most transcript sites with 2,352.

In previous studies of pear [24], 186 conserved pear miRNAs and 37 miRNA families were identified. Comparatively, there were 21 of the same miRNA families (miR156, miR157, miR164, miR166, miR167, miR168, miR172, miR2111, miR319, miR390, miR394, miR395, miR396, miR397, miR398, miR399, miR408, miR4414, miR477, miR535, miR827) found in our research. Additionally, miR156 was shown to be the largest miRNA family in pear. In Japanese apricot [15] and peanut [31], miR156 was also found to be the largest miRNA family of all the identified miRNA families. The result may suggest the fundamental function played by miR156 across various species.

The analysis of read counts for known miRNA families suggested the great expression frequency divergences among these miRNAs. With respect to the abundance of each miRNA family, the frequencies varied from 2 to 368,116 reads, indicating that expression varies significantly among different miRNA families. In the six libraries, miR165a showed the highest sequencing reads, which may suggest the largest expression in pear fruit among miRNA families. The varied abundance is also identified in certain members from the same family. For example, the abundance of miR167 varied from 8 to 4,598 reads in 110 DAF library, as was the case for some other families, including miR156 (6 to 70,015 reads) and miR164 (45 to 28,900 reads). The two members of the miR4414 family showed distinct sequencing reads, miR4414a with 33,340 and miR4414a* with 2 in 15 DAF library. The two members of the miR164 family also observed the similar phenomenon at the 15 DAF library, miR164a showed abundance with 46,332 and miR164c* with 33. The results indicate that expression levels of different members within one miRNA family varied greatly during fruit development. In addition, the multiple locations for the same miRNA varieties also suggests there were many transcript sites for each miRNA in the genome, so there was a choice of member to transcribe in the same miRNA family, which may explain the expression variation.

### Identification of novel miRNAs in pear fruit

**Figure 2:**
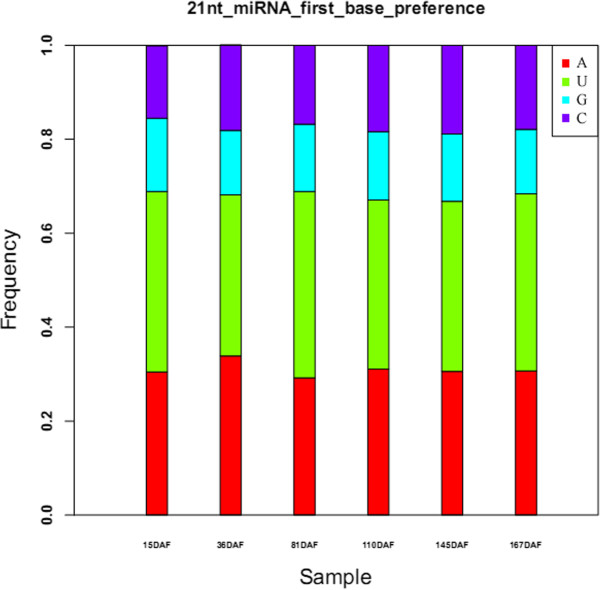
**First base bias at 21 nt of novel pear miRNA.** Y-axis represents the frequency of nucleotides and X-axis represents different libraries. Four different colors in the bars represent the four kinds of nucleotide.

MIREAP software was used to predict novel miRNAs in fruit by exploring the secondary structure compared with whole genome data [23]. 178,944 total reads and 654 unique reads were identified as novel miRNAs. There were 297 novel miRNAs in total and 41 of them classified into 16 different known miRNA families. Precursors forming hairpin structures were obtained. The average negative minimal folding free energy (MFE) was -51.48 kcal/mol (Additional file 2), which was similar to the sequencing data for maize miRNA precursors (-61.15 kcal/mol) [10] and *Arabidopsis thaliana* miRNA precursors (-57 kcal/mol) [4]. The length of the novel miRNA precursors was identified to range from 64 to 374 nt, which was quite close to Japanese apricot [15]. As previous reported plant miRNA prefers the length 21 nt with the 5′ end base bias to uracil (U) [32]. In our research, we also found base biases of 21 nt of novel miRNA showing the similar pattern, the U nucleotide took the leading percentage (37.05%), followed by A (31%), G (17.6%) and C (14.35%) in the six libraries (Figure 2).

### Validation of microRNA expression by qRT-PCR

**Figure 3:**
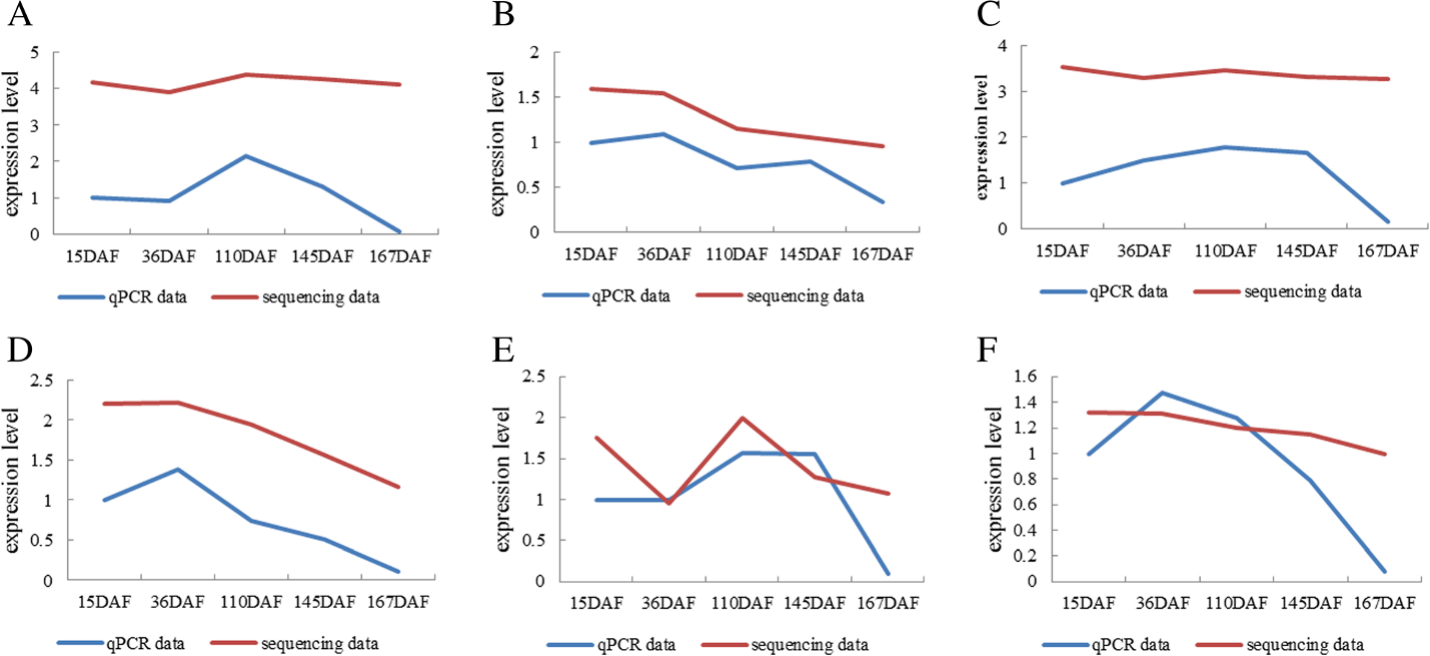
**Expression pattern confirmed by qRT-PCR and comparison with sequencing data.** Relative expression pattern of six different conserved miRNAs among various libraries are confirmed by qRT-PCR results by 2^-∆∆Ct^ method. For visualization, log_10_ method is applied to compute the TPM data of sequencing results. The paragraph **A to F** represent the expression profiles of miR166a, miR4376-5p, miR156k, miR396b-3p, miR164c* and miR159b-3p in sequence. Y-axis represents the expression level and X-axis represents different libraries. Blue lines represent the q-PCR data and red lines represent the sequencing data. The line charts indicate the similar expression patterns with miRNA sequencing.

In our research, qPCR was adopted to validate the sequencing results of six miRNAs (miR166a, miR4376-5p, miR156k, miR396b-3p, miR164c*, miR159b-3p), and was able to show that these miRNA are really expressed, and the changes during the fruit development are real. The line charts indicate similar expression patterns as shown in Figure 3, although the values of miRNA expression detected by two methods varied to some extent. So, the qPCR results confirmed the reliability and expression patterns of microRNA involved in pear fruit development through high throughput sequencing.

### MiRNAs mapped on pear genome reference

Using SOAP2 software, more than 15 million clean reads (69.335%) were perfectly mapped to the pear genome [23]. Compared with 297 miRNAs identified in pear genome [18], there were 15 newly discovered conserved miRNA families which may suggest expression specifically in pear fruit, including miR156, miR157, miR160, miR166, miR172, miR2118, miR2590, miR319, miR395, miR399, miR4376, miR4414, miR447, miR479, and miR827. Among them, miR4376 has been suggested to regulate transgenic tomato flower morphology and fruit yield by targeting Ca^2+^-ATPase, SI *ACA10* [33]. MiR2118 was not detected in the libraries derived from seedlings, root apices and leaves, but could be detected in libraries derived from early development stages of maize anthers [34, 35]. Johnson et al. [9] proposed that miR2118 regulated the development of reproductive structures in cereals. Previous research indicated that these miRNAs showed tissue-specific expression pattern and may play significant roles in plant specific tissue development. However, accurate roles for these miRNAs in pear fruit still need further experimental verification.

When mapping the miRNAs against pear genome reference sequences [23], 266 out of the 362 pear miRNAs could be identified on ~512Mbp pear genome. The mapping result showed that a single miRNA might have more than one homolog. In the Additional file 3, data showed that all 17 chromosomes had miRNAs distribution on both sense and anti-sense strands, but with uneven distribution of miRNAs. The sense and anti-sense strands of Chr15 had the greatest number of reads, while Chr1 had the least number of reads. The results indicated that miRNAs have uneven distribution on different chromosomes of pear.

MiRNAs were proven to present in clusters. In human, the mir17 cluster contains six precursor miRNAs within about 1 kb on chromosome 13 [36]. Meanwhile, 25.35% in *Arabidopsis thaliana*, 17.09% in *Populus trichocarpa*, 22.29% in *Oryza sativa* and 21.62% in *Sorghum bicolor* of miRNAs have been found to cluster within a 10-kb region in previous studies [37]. In this study, we found 14 miRNA clusters at distances of 10 kb and 66 clusters at 100 kb (Additional file 4). The clustering phenomenon is distributed on different regions of the genome. Furthermore, different miRNAs in the same cluster were shown to keep similar expression patterns, as previously reported [38]. Thus the targets of miRNAs in the cluster were shown to have the same function. MiRNA genes are clustered in the genome with an arrangement and expression pattern implying transcription as a multi-cistronic primary transcript. Li et al. suggested that the co-transcription of similar or identical miRNAs in clusters for plants may be involved in gene dosage effect [39]. When compared with previous studies in microRNA identification of pear by Niu et al. [24], no similar clusters were found.

### MiRNAs comparison across pear and other plant species

MiRNAs in various plants, including several important fruit crops have been identified with next-generation sequencing technology. For instance, in apple, a total of 165 miRNAs were identified according to The Apple Gene Function & Gene Family DataBase v1.0 http://www.applegene.org/mirna.asp. In another *Rosaceae* plant, peach, 117 conserved miRNAs and 186 novel miRNA candidates have been identified [40]. In our analysis, 362 conserved miRNAs and 297 novel miRNAs were identified in pear, respectively. The variation of miRNAs in our research can give insight to widely existing highly-conserved miRNAs and can give a basis to further deep bioinformatics studies for miRNAs in pear, for instance the SNP identification of miRNAs.

The conservation and divergence of miRNAs among various plant species has been widely reported [41, 42]. In Jones-Rhoades’ study, miR156/157, miR172 and miR170/171 were regarded as highly conserved miRNAs, with orthologs in more than ten different plant families, while miR163 and miR158 were identified as non-conserved miRNAs [41]. In the perspective of Zhang et al. [7], the varied conservation of miRNAs suggests the multiple functions of miRNAs in plant development, and the miRNAs with low conservation may have species-specific characteristics in plant development.

**Figure 4:**
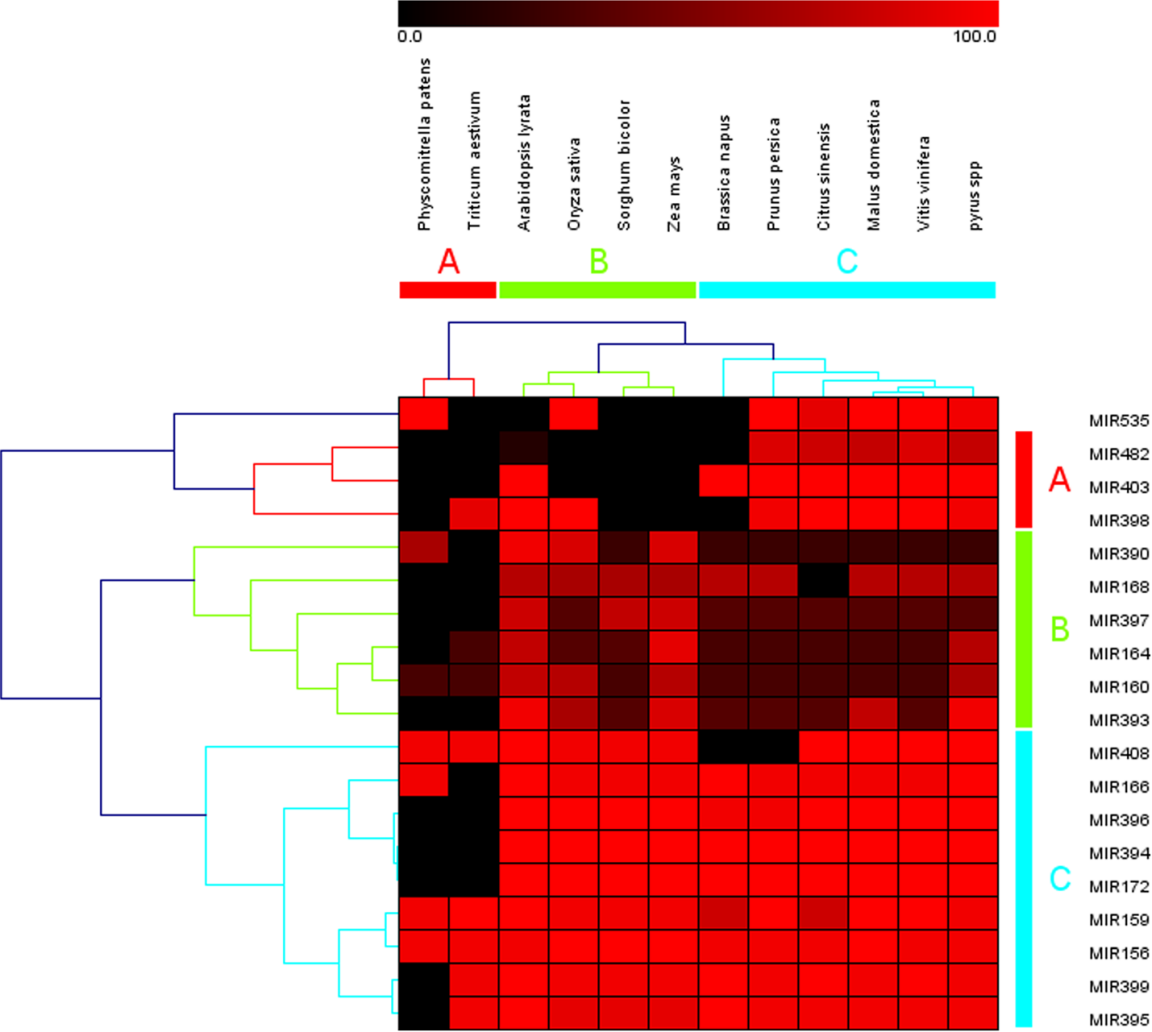
**Nineteen conserved miRNAs from pear fruit and their homologs in 11 other plant species.** The color shows the homology of miRNAs among the twelve plants. As the color gets closer to bright red, the relationship of the species gets closer to each other.

To study the conservation and evolution of the known miRNAs in pear fruit, comparisons were conducted among eleven other species including *Physcomitrella patens*, *Triticum aestivum*, *Arabidopsis lyrata*, *Oryza sativa*, *Sorghum bicolor*, *Zea mays*, *Brassica napus*, *Prunus persica*, *Citrus sinensis*, *Malue domestica,* and *Vitis vinifera* (Figure 4). Based on the analysis, several miRNAs showed a high level of conservation among various plant species, including miR395, miR399, miR156, miR159, miR172, miR394, miR396 and miR166, as more than ten plant species showed high sequence homology for these miRNAs. As expected, *Pyrus* and *Malus domestica* shared more than thirteen common conserved miRNA families, exhibiting higher conservation of miRNA families and high homology between the two species. On the converse, with less than five homologous miRNAs, *Physcomitrella patens* showed narrow homology relationship with *Pyrus*. The phenomenon can be explained by speciation of plants which was accompanied with the specialization of miRNAs playing species-specific roles [35, 42].

### Analysis of differentially expressed miRNAs

Differentially expressed miRNAs were identified by comparing the sequencing reads with significant correlations (*P* <0.05 and *P* <0.01) between the six libraries. Taking 15 DAF as control, 42 to 149 miRNAs had significant changes among different libraries. Among those miRNAs, there were two (miR839*, miR1871) and twelve miRNAs (miR1087, miR1310, miR169j*, miR2916, miR2936, miR319e, miR3627, miR395a, miR397a, miR398b, miR408, miR408b*) showing an increased or decreased abundance, respectively, from 15DAF to 167DAF. Additionally, the fluctuation patterns of mature miRNAs during the developmental stages were observed, all 362 conserved miRNAs were clustered by their expression patterns (Additional file 5). Taking the early stage (15DAF) as control, there were groups of miRNAs showing higher expression levels at later stages (36DAF to 167DAF), such as miR808, miR1028a-5p, miR1870-3p, miR5255, and miR5253. On the contrary, several miRNAs showed a gradually decreasing expression accompanying fruit development, including miR398a*, miR5245, miR5065, miR413, miR390a, miR167, miR393, miR164, and miR160. Thus, the differential expression patterns of miRNA for different fruit developmental stages in pear were confirmed in this study. The difference in expression profiles may suggest the relationship between miRNAs and pear fruit development. At specific developmental stages, different miRNAs may have the same expression pattern, which may lead to their co-regulatory role in fruit development. As for apple [43], compared with conserved miRNAs, novel miRNAs exhibited lower expression levels in pear.

**Figure 5:**
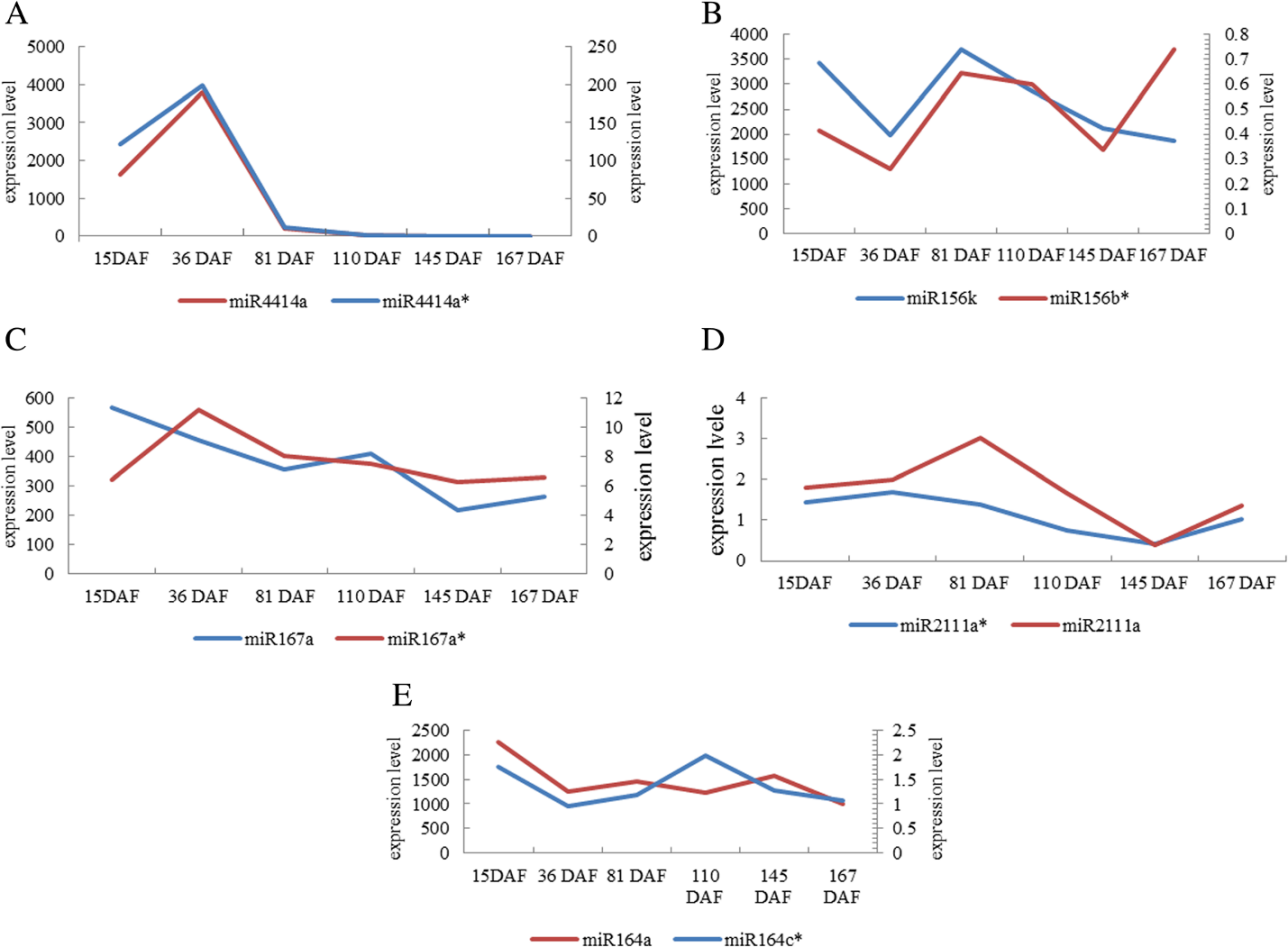
**Expression profiles of members in miRNA families.** Y-axis represents the expression level of miRNAs and X-axis represents different libraries. **A-E** represent sequencing reads of different members in five conserved miRNA families including miR4414a/4414a*, miR156k/156b*, miR167a/167a*, miR2111a*/2111a and miR164a/164c*). Similar expression patterns of different members in the miRNA family throughout development are shown.

Further analysis showed that different members in the same miRNA family present similar expression profiles, except in a few stages (Figure 5), while the expression patterns between different miRNA families varied. In the study of peanut [31], the same results were also concluded. Two members of the ahy-miR159 family were similar and this may provide valuable information on the role that miRNAs play in plant growth [31].

MiRNA cluster analysis showed that members of a given cluster have parallel expression patterns, which implies that miRNA cluster members coordinately regulate fruit development. It was noticeable that different clusters contained quite similar miRNAs while the locations of clusters varied. The phenomenon suggests that those miRNAs are always transcribed together and may be transcribed from different chromosomes or different segments on the same chromosome. A similar phenomenon has been previously reported [44]. The *mir-3* to *mir-6* genes were clustered and showed high similarity in expression profiles [45].

### Target gene analysis of miRNAs in pear fruit

**Figure 6:**
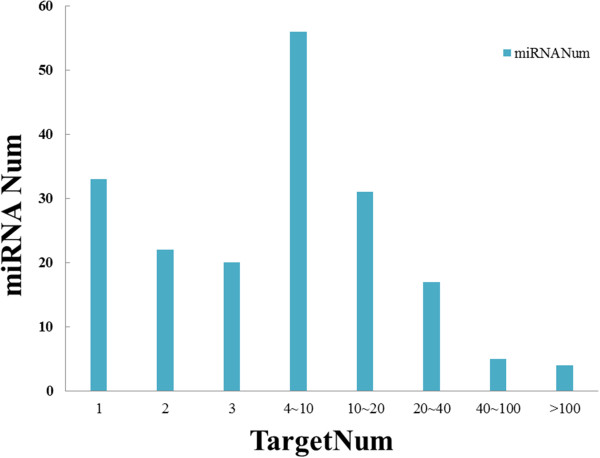
**The distribution of target gene numbers for each conserved miRNA among developmental stages.** X-axis represents the interval for the target gene number of each conserved miRNA. Y-axis represents the number of miRNA in each interval.

MicroRNA regulates gene expression through inhibiting translation or degrading mRNA at a certain site [46]. Currently, bioinformatics methods have been used to decipher target genes in several studies [47, 48]. To better understand the roles pear miRNAs play in fruit development, PickPlantTar was used to predict potential miRNA target genes by using the most abundant mature miRNA as queries. By a stringent criteria described in the methodology, 2,216 targets for 188 known miRNAs and 1,127 targets for 184 novel miRNAs with differential expression patterns were predicted (Additional file 6). The number of putative target genes for each conserved miRNA varied from 1 to 226. As showed in Figure 6, more than 50 miRNAs targeted 4 to 10 genes. Thirty-three miRNAs had only one target gene, which may suggest a unique regulatory function played by these miRNAs. Meanwhile, we found that 4 miRNAs had more than 100 predicted target genes. For instance, miR396b targeted 226 genes, the highest among the known miRNAs, followed by miR5564 and miR4993 with 149 and 137 target gene numbers, respectively, which might indicate the wide regulation functions of these miRNAs. In addition, as suggested by Bonnet et al. [49], highly repetitive motifs corresponding to the miRNA sequence may contribute to the ubiquity of target genes, which means the possibility of the potential false prediction. Further experiments are needed to confirm the accurate function for these miRNAs.

**Figure 7:**
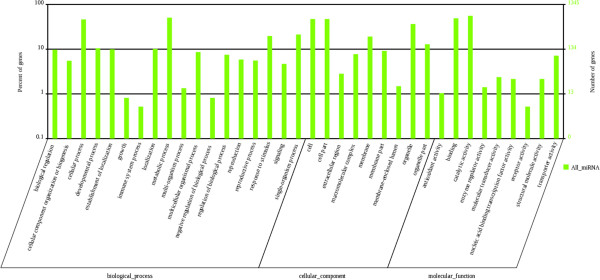
**GO annotation of target genes.** Y-axis (left) represents percentages of genes identified in this study, Y-axis (right) represents the actual gene number. The genes were annotated in three main categories including biological progress, cellular component, and molecular function (X-axis).

The 2,216 targets for 188 known miRNAs are predicted to be implicated in a broad range of physiological or biochemical processes. All annotated targets were BLASTX searched against the protein database, followed by Gene Ontology (GO) analysis to assess the genes’ putative functions. GO analysis contained three ontologies: biological process, cellular component, molecular function [50]. Shown as results of Figure 7, 18 categories in biological process were classified by the genes, followed by 9 categories for cellular component and molecular function, respectively. As metabolic processes are the most active process in fruit development, it is understandable that cellular processes and metabolic processes were the top two GO terms within biological processes. On the side of molecular function, the binding term and catalytic activity term occupied the largest numbers of genes. The result may suggest that miRNAs play key roles in regulation of substance metabolism because enzymes are indispensable catalysts for fruit development.

### MiRNAs regulate pear fruit development

**Figure 8:**
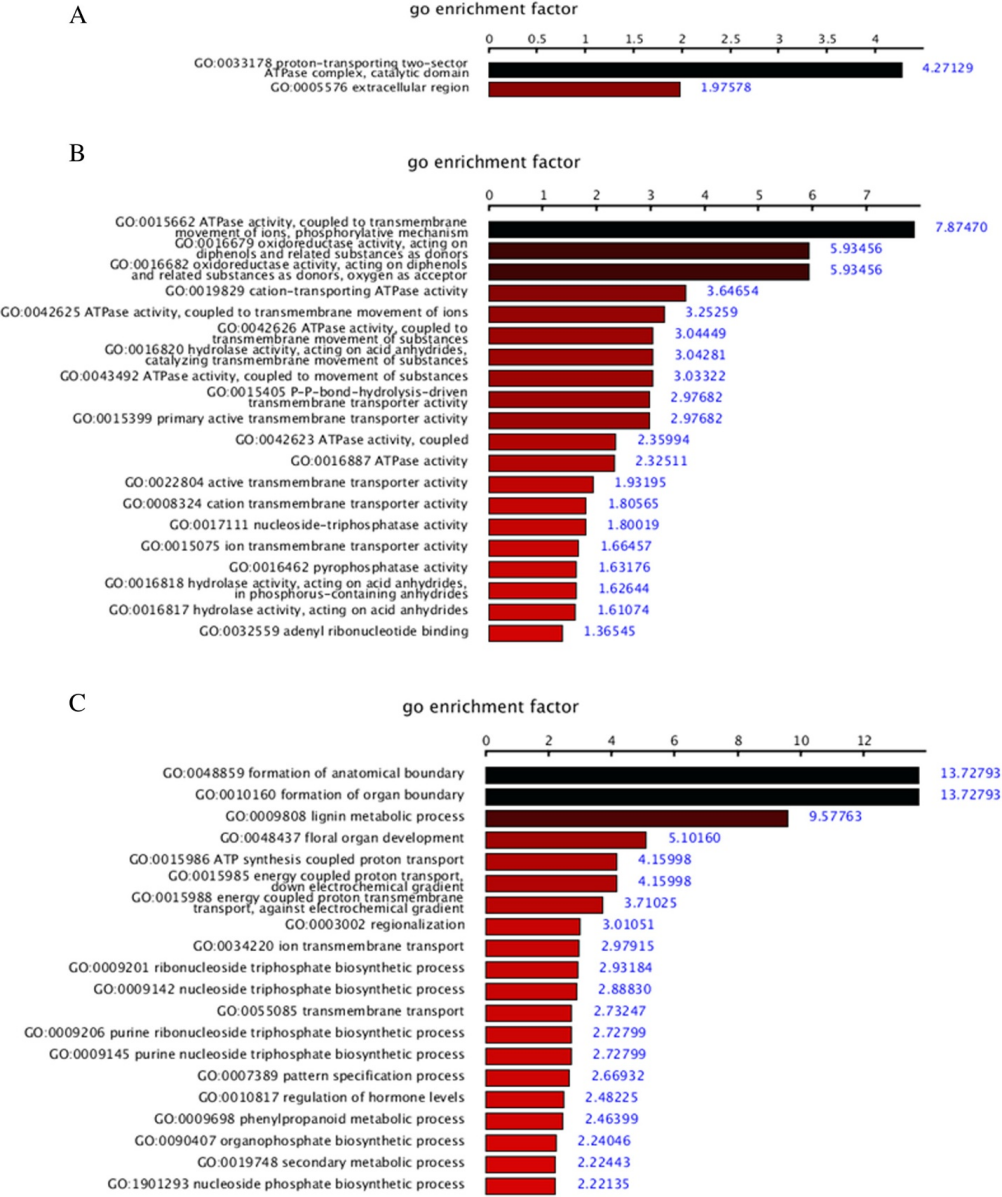
**Target gene GO enrichment factor screened by the p-value. A**, **B** and **C** represent three ontologies: cellular component, molecular function and biological progress respectively.

The development of pear fruit is a complex process, as major changes are involved in various metabolic pathways [51]. In order to uncover the role of miRNA in regulating the fruit growth and maturity, the target genes of miRNAs were identified by Kyoto Encyclopedia of Genes and Genomes (KEGG) pathways according to the *P*-value and enrichment factor. A total of 254 pathways were illustrated as a result of a BLAST search against the KEGG database (Additional file 7). Screening by the criteria described in the methods, we assessed GO terms ordered by the enrichment factor (Figure 8). In the category of molecular function, the ATPase activity had the highest GO enrichment factor, 7.87470.

It can be concluded (Table 2) that the targets of conserved miRNAs are involved in all parts of plant development, from plant hormone signal transduction (Pbr042396.1) to plant pathogen interaction (Pbr041100.1). Besides the universal target genes such as ARF and UDP-sugar phrophosphorylase, unusual targets were also identified. The first class of targets suggested that the miRNAs take part in basic pear fruit metabolism, while the second class of targets showed the species-specific role played by miRNAs in pear fruit.

**Table 2:** Target genes of conserved miRNAs (excerpted*)

microRNA	Target gene ID	Gene annotation/pathway (p-value)
miR1061-3p	Pbr041100.1	Plant-pathogen interaction(7.190677e-06)
	Pbr025269.1	Plant-pathogen interaction(7.190677e-06)
	Pbr019706.1	Plant-pathogen interaction(7.190677e-06)
	Pbr014433.1	Plant-pathogen interaction(7.190677e-06)
	Pbr010237.1	Plant hormone signal transduction(0.01510044) Plant-pathogen interaction(7.190677e-06)
	Pbr002521.1	Plant hormone signal transduction(0.01510044) Plant-pathogen interaction(7.190677e-06)
	Pbr029017.1	Phenylalanine, tyrosine and tryptophan biosynthesis(1.488743e-06) Metabolic pathways(0.7902142) Other glycan degradation(0.855243) Fructose and mannose metabolism(0.6615606) Glycosphingolipid biosynthesis - ganglio series(0.5541742) Sphingolipid metabolism(0.1582752) Lysosome(0.9793836) Amino sugar and nucleotide sugar metabolism(0.6427769) Glycosaminoglycan degradation(0.4927528) Ribosome(0.987106) Galactose metabolism(0.2583273) Biosynthesis of secondary metabolites(0.9498542)
	Pbr031602.1	Mineral absorption(0.00709937)
	Pbr029046.1	Mineral absorption(0.00709937)
	Pbr021467.1	Mineral absorption(0.00709937)
	Pbr002476.1	Mineral absorption(0.00709937)
	Pbr012616.2	Metabolic pathways(0.7902142) Folate biosynthesis(0.1157641) ABC transporters(0.06561596) Arginine and proline metabolism(0.1871821) GABAergic synapse(0.9817247) Nitrogen metabolism(0.5698157) Polyketide sugar unit biosynthesis(0.3271947) Alanine, aspartate and glutamate metabolism(0.9892745) Glyoxylate and dicarboxylate metabolism(0.9626975) Glutamatergic synapse(0.9998555) Streptomycin biosynthesis(0.5326464) Microbial metabolism in diverse environments(0.9297261) Biosynthesis of secondary metabolites(0.9498542) Two-component system(0.2053418)
	Pbr021166.1	Influenza A(0.2265907) Tuberculosis(0.08865112) Leishmaniasis(0.04564140) Chagas disease (American trypanosomiasis)(0.02067325) Plant-pathogen interaction(7.190677e-06) Toxoplasmosis(0.2633427) Toll-like receptor signaling pathway(0.05315244) Neurotrophin signaling pathway(0.05126187) Apoptosis(0.06810953) NF-kappa B signaling pathway(0.073712) Measles(0.06773311) Pertussis(0.03473645)
	Pbr040822.1	Flavonoid biosynthesis(0.599654) Metabolic pathways(0.7902142) Biosynthesis of secondary metabolites(0.9498542)
	Pbr040817.1	Flavonoid biosynthesis(0.599654) Metabolic pathways(0.7902142) Biosynthesis of secondary metabolites(0.9498542)
	Pbr021205.1	Ethylbenzene degradation(0.369114) Leishmaniasis(0.04564140) Chagas disease (American trypanosomiasis)(0.02067325) Plant-pathogen interaction(7.190677e-06) Toxoplasmosis(0.2633427) Neurotrophin signaling pathway(0.05126187) Microbial metabolism in diverse environments(0.9297261) NF-kappa B signaling pathway(0.073712) Measles(0.06773311) Tyrosine metabolism(0.3092827) Influenza A(0.2265907) Aminobenzoate degradation(0.3980524) Tuberculosis(0.08865112) Metabolic pathways(0.7902142) Fructose and mannose metabolism(0.6615606) Limonene and pinene degradation(0.656067) Benzoate degradation(0.3951967) Toll-like receptor signaling pathway(0.05315244) Apoptosis(0.06810953) Pertussis(0.03473645) Pyrimidine metabolism(0.0004838167)
	Pbr035730.1	Cytosolic DNA-sensing pathway(6.107676e-13) Metabolic pathways(0.7902142) Plant-pathogen interaction(7.190677e-06) RNA polymerase(1.034169e-09) Pyrimidine metabolism(0.0004838167) Epstein-Barr virus infection(8.479755e-05) Huntington’s disease(8.38133e-08) Purine metabolism(0.0001980336)
	Pbr010706.1	Circadian rhythm - mammal(0.3646747) Wnt signaling pathway(0.3688827) Protein processing in endoplasmic reticulum(0.9999496) TGF-beta signaling pathway(0.9755566) Ubiquitin mediated proteolysis(0.321184) Oocyte meiosis(0.9154166) Cell cycle(0.9997656) Cell cycle - yeast(0.5227999) Herpes simplex infection(0.9478489)
	Pbr026497.1	Cell cycle - yeast(0.5227999)
miR1121	Pbr018590.1	Metabolic pathways(0.7902142) Glycosylphosphatidylinositol(GPI)-anchor biosynthesis(0.1935023)
miR1318	Pbr027745.1	Valine, leucine and isoleucine degradation(0.853519) Propanoate metabolism(0.7921382) Metabolic pathways(0.7902142) Lysine biosynthesis(0.3398862) Glycine, serine and threonine metabolism(0.8336738) Tryptophan metabolism(0.4881696) Arginine and proline metabolism(0.1871821) Pyruvate metabolism(0.9296067) Glycolysis / Gluconeogenesis(0.9894463) beta-Alanine metabolism(0.8613309) Fatty acid metabolism(0.830646) Histidine metabolism(0.8285719) Glycerolipid metabolism(0.707723) Lysine degradation(0.3979612) Biosynthesis of secondary metabolites(0.9498542) Ascorbate and aldarate metabolism(9.178918e-07)
	Pbr009875.1	Spliceosome(0.338066)
	Pbr020008.1	Regulation of autophagy(0.2863020)
	Pbr013669.1	Plant-pathogen interaction(7.190677e-06)
	Pbr009166.1	Plant hormone signal transduction(0.01510044)
	Pbr036152.1	Phenylpropanoid biosynthesis(0.1081946) Metabolic pathways(0.7902142) Biosynthesis of secondary metabolites(0.9498542) Phenylalanine metabolism(0.1547410)
	Pbr038845.1	Naphthalene degradation(0.930259) Aminobenzoate degradation(0.3980524) Chloroalkane and chloroalkene degradation(0.9450628) Polycyclic aromatic hydrocarbon degradation(0.4501106) Microbial metabolism in diverse environments(0.9297261) Bisphenol degradation(0.8789056) Limonene and pinene degradation(0.656067) Biosynthesis of secondary metabolites(0.9498542)
	Pbr015038.1	Metabolic pathways(0.7902142) Citrate cycle (TCA cycle)(0.997454) Microbial metabolism in diverse environments(0.9297261) Biosynthesis of secondary metabolites(0.9498542)
	Pbr031471.1	GABAergic synapse(0.9817247)
	Pbr013295.1	Circadian rhythm - plant(0.924008)
	Pbr026842.1	Alanine, aspartate and glutamate metabolism(0.9892745) Bacterial secretion system(0.8448431) Metabolic pathways(0.7902142) Protein export(0.6659996) Pyrimidine metabolism(0.0004838167)
	Pbr004124.1	Alanine, aspartate and glutamate metabolism(0.9892745) Bacterial secretion system(0.8448431) Metabolic pathways(0.7902142) Protein export(0.6659996) Pyrimidine metabolism(0.0004838167)
miR160a	Pbr042396.1	Plant hormone signal transduction(0.01510044)
	Pbr039066.1	Plant hormone signal transduction(0.01510044)
	Pbr037271.1	Plant hormone signal transduction(0.01510044)
	Pbr016467.1	Plant hormone signal transduction(0.01510044)
	Pbr008550.1	Plant hormone signal transduction(0.01510044)
	Pbr008404.1	Plant hormone signal transduction(0.01510044)
	Pbr008352.1	Plant hormone signal transduction(0.01510044)
	Pbr008195.1	Plant hormone signal transduction(0.01510044)

*More target genes of all miRNAs are listed in Additional file 6.

**Figure 9:**
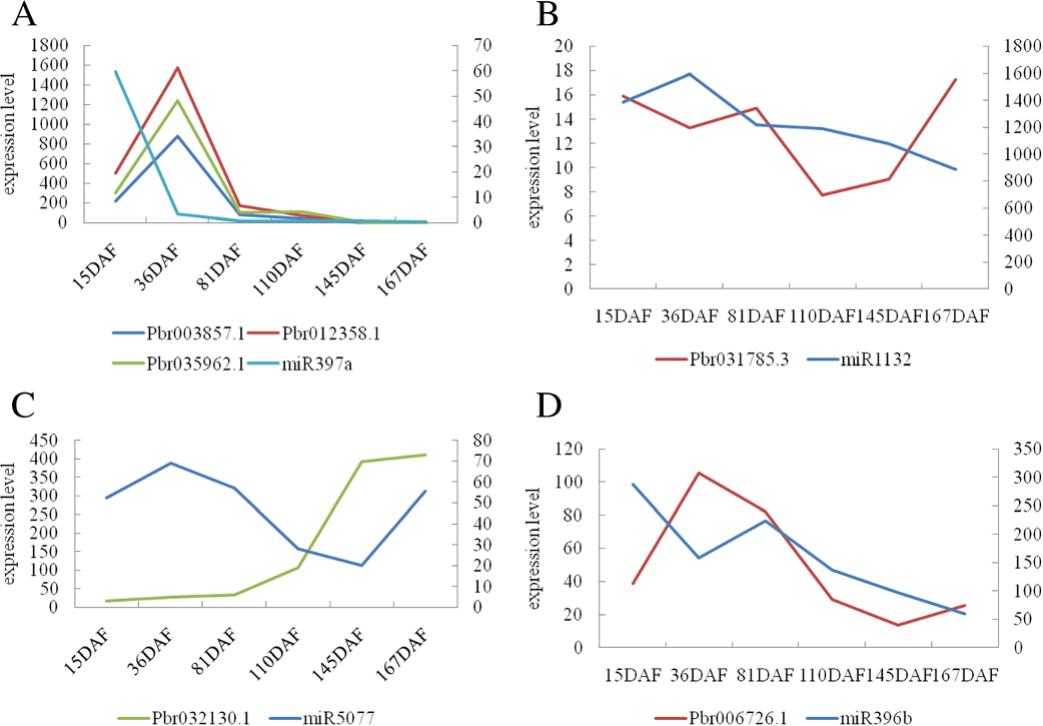
**Expression pattern of miRNAs and target genes. A**, **B**, **C**, and **D** represent the comparisons of the expression patterns during pear fruit development for miR397a, miR1132, miR5077 and miR396b and their putative target genes, respectively. Y-axis (left) represents expression level of target genes, Y-axis (right) represents the expression level of miRNAs.

When we further explored the relationship of some miRNAs and their putative target genes (RNAseq data from pear genome project [23]), it was found that an opposite expression pattern presented in different stages of fruit development (Figure 9), such as miR397a, miR1132, miR5077 and miR396b, which suggested miRNAs were involved in lignin, sugar and acid pathways of fruit development. Hence, we took a deeper look at the miRNAs identified that are involved in several important pear fruit development metabolic pathways, including lignin pathway, sugar and acid pathway, hormone signaling and carbohydrate metabolism activities.

### Putative miRNAs related with pear fruit lignin pathway

Stone cells are one of the important features of fruit quality in pear, rarely seen in other fresh fruit. As the primary component of stone cells, lignin synthesis has direct impact on their production. Lignin is an aromatic heteropolymer derived from phenylpropanoid [52]. Previously, there were reports that lignin may improve plant resistance to pathogens to a certain degree [53]. In Zinnia [54], the CCoAOMT is involved in the alternative methylation pathway in lignin biosynthesis. In *Arabidopsis* [55], overexpression of SND1, a NAC domain transcription factor, results in activation of the biosynthetic genes for secondary wall. In pear, Wu et al. reported 66 lignin synthesis-related gene families and depicted their pathways [23]. The RNA-seq data for these genes showed that they are highly expressed in the early stages, almost 10-fold higher than near ripening.

In poplar, it has been demonstrated that overexpressed Corngrass1 miRNA, belonging to the miR156 family, in the transgenic poplar plant 35S:Cg1 has dramatic effects on plant architecture and stem lignin content [56]. The induction of miR828 in sweet potato may regulate the phenylpropanoid pathway and biosynthesis of lignin by repressing the expression of *IbMYB* and *IbTLD* [57].

**Figure 10:**
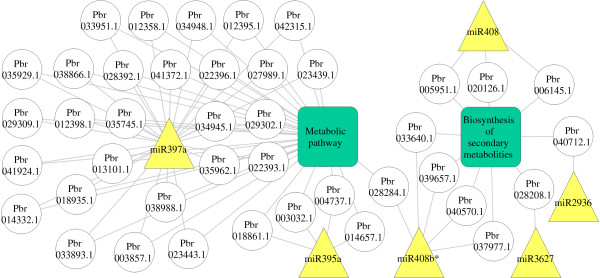
**Relationship of miRNAs and target genes in lignin pathway.** The triangles, boxes and circles represent miRNAs, pathways and target genes respectively.

Through GO annotation of the target genes and comparison with KEGG database, six miRNAs (miR397a, miR395a, miR408, miR2936, miR408b*, and miR3627) among the twelve down-regulated miRNAs were shown involving in metabolic pathway and biosynthesis of secondary metabolites comprising the lignin synthesis pathway (Figure 10). Among them, 27 target genes of miR397a were shown to be the most significant miRNAs involved in the lignin pathway. These genes (Table 3) were the homologs of *Ptr-lac* or *Ath-lac*. The laccase-like enzyme could catalyze coniferyl alcohol polymerization, implicating laccases in lignification [52]. The transcripts per million (TPM) value for miR397a showed a decrease in the development periods, consistent with the role of pear fruit lignin synthesis showed by Wu et al. [23], while the transcription of several targets showed the opposite tendency from miR397a (Figure 9).

**Table 3:** **MiRNAs and target genes involved in lignification and sugar and acid pathways**

miRNA name	Target gene ID	Target gene description	Pathway
miR397a	Pbr003857.1	Laccase	Metabolic pathways(0.7902142)
	Pbr012358.1		Metabolic pathways(0.7902142)
	Pbr012395.1		Metabolic pathways(0.7902142)
	Pbr012398.1		Metabolic pathways(0.7902142)
	Pbr013101.1		Metabolic pathways(0.7902142)
	Pbr014332.1		Metabolic pathways(0.7902142)
	Pbr018935.1		Metabolic pathways(0.7902142)
	Pbr022393.1		Metabolic pathways(0.7902142)
	Pbr022396.1		Metabolic pathways(0.7902142)
	Pbr023439.1		Metabolic pathways(0.7902142)
	Pbr023443.1		Metabolic pathways(0.7902142)
	Pbr027989.1		Metabolic pathways(0.7902142)
	Pbr028392.1		Metabolic pathways(0.7902142)
	Pbr029302.1		Metabolic pathways(0.7902142)
	Pbr029309.1		Metabolic pathways(0.7902142)
	Pbr033893.1		Metabolic pathways(0.7902142)
	Pbr033951.1		Metabolic pathways(0.7902142)
	Pbr034945.1		Metabolic pathways(0.7902142)
	Pbr034948.1		Metabolic pathways(0.7902142)
	Pbr035745.1		Metabolic pathways(0.7902142)
	Pbr035929.1		Metabolic pathways(0.7902142)
	Pbr035962.1		Metabolic pathways(0.7902142)
	Pbr038866.1		Metabolic pathways(0.7902142)
	Pbr038988.1		Metabolic pathways(0.7902142)
	Pbr041372.1		Metabolic pathways(0.7902142)
	Pbr041924.1		Metabolic pathways(0.7902142)
	Pbr042315.1		Metabolic pathways(0.7902142)
miR1132	Pbr031785.3	Malate_PEPCK_PEPCK-ATP	
miR1318	Pbr015038.1	Citrate_ACL_ACLY	Metabolic pathways(0.7902142),Microbial metabolism in diverse environments(0.9297261),Biosynthesis of secondary metabolites(0.9498542),Citrate cycle (TCA cycle)(0.997454)
miR2635	Pbr038213.1	Malate_PEPC_PEPC	Carbon fixation in photosynthetic organisms(0.7361554),Metabolic pathways(0.7902142),Methane metabolism(0.9082858),Pyruvate metabolism(0.9296067),Microbial metabolism in diverse environments(0.9297261),Carbon fixation pathways in prokaryotes(0.9871138)
miR394a	Pbr020930.1	Malate_MalT	
	Pbr023085.1	Malate_MalT	
miR396b	Pbr006726.1	DNA_G6PDH_G6PDH	Metabolic pathways(0.7902142),Microbial metabolism in diverse environments(0.9297261),Biosynthesis of secondary metabolites(0.9498542),Pentose phosphate pathway(0.975254),Glutathione metabolism(0.9974516)
	Pbr010337.1	Malate_MalT	
	Pbr016769.1	Malate_MalT	
	Pbr016770.1	Malate_MalT	
miR5077	Pbr023965.1	Sugar_HT	
	Pbr032130.1	Sugar_HT	Meiosis - yeast(0.9924183)
	Pbr033292.1	Sugar_HT	
miR5500	Pbr029752.1	Sugar_HK_HK	Galactose metabolism(0.2583273),Butirosin and neomycin biosynthesis(0.4998806),Streptomycin biosynthesis(0.5326464),Amino sugar and nucleotide sugar metabolism(0.6427769),Fructose and mannose metabolism(0.6615606),Metabolic pathways(0.7902142),Insulin signaling pathway(0.878923),Carbohydrate digestion and absorption(0.919634),Microbial metabolism in diverse environments(0.9297261),Biosynthesis of secondary metabolites(0.9498542),Starch and sucrose metabolism(0.9606751),Glycolysis / Gluconeogenesis(0.9894463),Type II diabetes mellitus(0.9906034)
miR825*	Pbr016798.1	Sugar_ALD-ALD1	Phenylalanine, tyrosine and tryptophan biosynthesis(1.488743e-06),Sulfur metabolism(0.04454147),Fructose and mannose metabolism(0.6615606),Carbon fixation in photosynthetic organisms(0.7361554),Metabolic pathways(0.7902142),Cysteine and methionine metabolism(0.9034785),Microbial metabolism in diverse environments(0.9297261),Biosynthesis of secondary metabolites(0.9498542),Pentose phosphate pathway(0.975254),Glycolysis / Gluconeogenesis(0.9894463)
miR952b	Pbr012392.1	Sugar_F16BP_F16BP1	Fructose and mannose metabolism(0.6615606),Carbon fixation in photosynthetic organisms(0.7361554),Metabolic pathways(0.7902142),Insulin signaling pathway(0.878923),Methane metabolism(0.9082858),Microbial metabolism in diverse environments(0.9297261),Biosynthesis of secondary metabolites(0.9498542),Pentose phosphate pathway(0.975254),Glycolysis / Gluconeogenesis(0.9894463)
	Pbr012401.1	Sugar_F16BP_F16BP1	Fructose and mannose metabolism(0.6615606),Carbon fixation in photosynthetic organisms(0.7361554),Metabolic pathways(0.7902142),Insulin signaling pathway(0.878923),Methane metabolism(0.9082858),Microbial metabolism in diverse environments(0.9297261),Biosynthesis of secondary metabolites(0.9498542),Pentose phosphate pathway(0.975254),Glycolysis / Gluconeogenesis(0.9894463)
	Pbr035932.1	Sugar_F16BP_F16BP1	Fructose and mannose metabolism(0.6615606),Carbon fixation in photosynthetic organisms(0.7361554),Metabolic pathways(0.7902142),Insulin signaling pathway(0.878923),Methane metabolism(0.9082858),Microbial metabolism in diverse environments(0.9297261),Biosynthesis of secondary metabolites(0.9498542),Pentose phosphate pathway(0.975254),Glycolysis / Gluconeogenesis(0.9894463)

The conclusion was consistent with the research in *Populus trichocarpa* [58]. Twenty-nine laccase genes were predicted to be targets of ptr-miR397a [59]. By over expression of *Ptr-miR397a* in the transgenic lines, 17 *PtrLACs* were down-regulated, which resulted in a ~40% decreasing of the total laccase activity, and lignin content was also reduced. The transcriptome-based gene expression implicated the ptr-miR397a and *lac* in co-regulating lignin biosynthesis in wood formation. The detailed function of miR397a in pear fruit lignification still needs further studies and confirmed biological experiments.

Besides the miRNAs with significant differential expression, several other miRNAs were also analyzed according to their target gene annotation. MiR3711, miR419, and miR5260 targeted the hydroxycinnamoyl transferase (*HCT*) gene, which is known to promote lignin synthesis [60]. According to analysis in the pear genome [23], *HCT* gene is one of the pivotal genes in synthesis of lignin in pear fruit, leading to high levels of conversion of p-coumaroyl-CoA (C3’H) into caffeoyl-Coa (PCC) and feruloyl-CoA (FC). Another two key genes analyzed in lignin synthesis peroxidase (POD) and 4CL, are putatively targeted by miR5021 and miR396b, respectively.

### Putative miRNAs related with pear fruit sugar and acid pathway

Pear fruit quality and flavor are largely impacted by the composition and content of sugar and acid, thus metabolism of sugar and acid is one of the most significant fruit development characteristics. KEGG pathway analysis showed 24 sugar and acid pathways in pear fruit development, such as fructose and mannose metabolism, galactose metabolism, carbon fixation in photosynthetic organisms, glycolysis/gluconeogenesis, and biosynthesis of secondary metabolites. By the annotation of targets, nine miRNAs were identified as related to genes taking part in the above pathways. As listed in Table 3, these include *Malate PEPCK-ATP* (Pbr031785.3, Pbr038213.1), *Citrate Acly*(Pbr015038.1), *Malate Malt* (Pbr020930.1, Pbr023085.1, Pbr010337.1, Pbr016769.1, Pbr016770.1).

From the differentially expressed miRNAs (Additional file 5), several miRNAs have higher expression level in early phases than in mature phases. In early development phases, miR4414a* showed higher TPM (121.5524 in 15DAF and 198.7602 in 36DAF). When the fruit reached the mature phase, the TPM gradually decreased (10.8494 in 81DAF, 0.7175 in 110DAF, 0.1273 in 145DAF, and 0.1649 in 167DAF). Dihydrolipoyl transacetylase (or E_2_, Pbr015262.1) was suggested to be the putative target of miR4414a*. E_2_ is one of the main enzymes of Tricarboxylic acid cycle (TAC) [61], which is known for its wide role in many pivotal plant metabolism pathways.

### Putative miRNAs regulated other pear fruit development genes

The majority of miRNA molecules chosen for this study and the complementary sites within their target genes display a high degree of conservation between species. Thus, the pear genome database was examined for the presence of potential miRNA targets. Auxin response factor (ARF), a transcription factors, regulates auxin signal transduction during plant growth. Niu et al. [24] previously reported putative orthologs of ARF transcription factors as targets of pear miR160 family. In our study, the same result was identified. MiR160a potentially targets *ARF16*, *ARF17* and *ARF18*. As a companion, miR167 was identified to target the hypothetical proteins LOC100242923 and LOC100265118. MiR160 and miR167 have been experimentally confirmed in several plant species including *Arabidopsis thaliana* [62] and soybean [63] that bind complementary sites in the UTR of ARF.

Known as a catalyzer of the conversion of various monosaccharide 1-phosphates to the respective UDP-sugars, UDP-sugar phrophosphorylase was shown to be essential in pollen development in *Arabidopsis* [64], and was putatively targeted by miR782 in our analysis. While there have previously been rare reports about miR782, it is necessary to do further studies to confirm its function.

## Conclusion

We identified 337 conserved miRNAs and 297 novel miRNAs for pear fruit at different developmental stages using the Illumina Hiseq2000 sequencing method. Through comparison with the pear genome, the transcript sites for these miRNAs were located. Fourteen miRNA clusters at 1 kb and 66 miRNA clusters at 10 kb were identified, respectively. Analysis of differential expression data suggested some roles for miRNAs in pear fruit development. Combined with target prediction and GO enrichment analysis, 2216 targets for 188 known miRNAs and 1,127 targets for 184 novel miRNAs were predicted and their functions shown. Several fruit development pathways were analyzed including lignin pathway, sugar and acid pathway, and hormone signaling. Combined with computational analysis and experimental confirmation, the research contributes valuable information for further functional research of microRNA in fruit development for pear and other species.

## Methods

### Plant materials

The fruits, 10–20 depending on the fruit size and period, of ‘Dangshansu’ were collected for each developmental stage 15, 36, 81, 110, 145, and 167 days after flowering (DAF), from three different trees that were grown in the pear depository at Pukou District pomology farm of Nanjing Agriculture University. After washing and removing kernels, fruit samples in the same stage were mixed and ground together, then immediately frozen in liquid nitrogen and stored at -80°C after collection.

### RNA extraction and deep sequencing

Total RNA was extracted from fruit samples using CTAB reagent according to the previously reported protocol by Chang et al. [65]. The concentration of RNA was measured by a NanoDrop 2000 spectrophotometer (NanoDrop Technologies, Wilmington, DE, USA) and visually ascertained with electrophoresis on a 1% agarose gel.

Six sRNA samples were constructed and sequenced by the Beijing Genomics Institute (BGI) (Shenzhen, Guangdong Province, China) using the Illumina HiSeq 2000 platform. The construction of the sRNA library consisted of the following steps: acrylamide gel purification of the RNA bands corresponding to a size range of 18–30 nt; ligation of the 3p and 5p adapters to the RNA in two subsequent steps, each followed by acrylamide gel purification; and a final step of PCR amplification to generate a cDNA colony template library for Illumina sequencing. The RNA-seq data used for the expression level of target genes was resourced from the published pear genome data which was available in the National Center for Biotechnology Information repository under accession PRJINA185970 (http://www.ncbi.nlm.nih.gov/bioproject/PRJNA185970).

### Bioinformatic analysis of miRNA identification

After sequencing, raw sequencing reads were processed into clean reads. During this procedure, we filtered out low quality, adapter contaminative tags and discarded the remaining reads with lengths smaller than 18 nt. All unique clean reads, particularly the non-redundant reads, were considered for further analysis, such as non-coding RNA identification and annotation. First of all, rRNA, scRNA, snoRNA, snRNA, and tRNA of clean reads were identified by BLASTall search against the Rfam (version 10.1) database. Next, in order to determine conserved miRNA, clean reads were compared with known miRNAs of plants deposited at miRBase 19.0. Finally, the other sequences that did not map to known miRNAs and other kinds of small RNA were referred to as un-annotated sequences for novel miRNA prediction. We identified potentially novel miRNA by MIREAP, and predicted the secondary structure by RNAfold. The criteria used for selecting miRNA were based on Meyers et al. [66], such as mature miRNAs having ~21 nt bases in length and miRNA precursor sequences being able to fold into a hairpin secondary structure.

### Quantitative real-time PCR validation

In order to confirm the sequencing results, qRT-PCR was applied to 6 miRNAs according to methods of previous research [67]. This method could not only confirm the existence of pear fruit miRNAs, but can also detect the expression level of miRNAs in various fruit development stages. Total RNA samples were sent to Oebiotech (Pudong district, Shanghai). The miScript II Reverse Transcription Kit (Qiagen) was used for miRNA synthesis. Briefly, reactions were completed in a volume of 20 μL, containing RNA templates 1 μg/Cone, 5× miScript HiSpec buffer 4 μL, 10 × nucleics mix 2 μL, miScript reverse transcriptase mix 2 μL and RNase free H_2_O up to 20 μL. The PCR conditions were performed in an ABI 9700 PCR system.

Based on the full miRNA sequences, the forward miRNA primers for real-time PCR were designed, with reverse primers the universal reverse primer for miRNA. The primer sequences are listed in Table 4. For each action, 1 μL cDNA was mixed with 2× SYBRReal PreMix (Tiangen) 5 μL, forward primers and reverse primers 0.2 μL (10 μM) respectively. Finally, RNase-free water was added up to the volume of 10 μL. PCR runs were 95°C for 10s, 60°C for 30s on the Roche LightCycler 480IIPCR system. Each reaction was repeated three times. The miRNA expression was quantified using the comparative ∆∆CT method [68]. All expression results were normalised to expression levels in the stem. 5S rRNAs [69] was used as an internal control.

**Table 4:** **qRT-PCR primer sequence list**

miRNA name	Forward primer sequence*
miR166a	TCGGACCAGGCTTCATTCCCC
miR4376-5p	ACGCAGGAGAGATGACGCCGA
miR156k	TTGACAGAAGAGAGTGAGCAC
miR396b-3p	TTCCACAGCTTTCTTGAACTG
miR164c*	TGGAGAAGCAGGGCACGTGCA
miR159b-3p	TGGATTGAAGGGAGCTCTACA
pear-5S	GAAAGATGCCAATTCATGCG

Note: *Reverse primers are universal reverse primers for miRNA.

### Differential expression analysis

The frequency of miRNAs in the two libraries was normalised to one million by the total number of miRNAs in each sample (transcripts per million (TPM) normalised expression = initial miRNA count*1,000,000/total count of clean reads). Following normalization, if the miRNA gene expression of two samples was zero, then it was revised to 0.01; if the miRNA gene expression of two samples was less than 1, owing to their too low expression, they were not included in analysis of differential expression.

The fold-change between different days after flowering and 15DAF was calculated as: fold-change = log_2_ (treat/15DAF). The *p-value* was calculated using the formula below.


### Prediction of target genes

The target genes for each miRNA were predicted as by the criteria of Allen et al. [70] and Schwab et al. [67], for miRNA targets of plants. Briefly, the criteria were as follows:

1. No more than four mismatches between miRNA and target (G-U bases count as 0.5 mismatches), 2) No more than two adjacent mismatches in the miRNA/target duplex, 3) No adjacent mismatches in positions 2–12 of the miRNA/target duplex (5′ of miRNA), 4) No mismatches in positions 10–11 of miRNA/target duplex, 5) No more than 2.5 mismatches in positions 1–12 of the miRNA/target duplex (5′ of miRNA).

### GO enrichment and KEGG pathway analysis

In order to further understand the functions of miRNA and their targets, GO enrichment and KEGG pathway analysis for all annotated miRNAs and their targets were performed. Blast2GO was employed to store information from the GO and KEGG pathway databases. All the sequences were identified by BLASTx searches against the GO protein database. A combined query was used in order to complete the GO annotation and pathway analysis against the GO and KEGG databases. Due to the large amount and complex branch structure of GO molecular functions, a significance threshold *P*-value was used to control the false discovery rate, referring to previous adjustment [71].

### Accession number

Sequencing data obtained in this research have been submitted to the NCBI under the accession number PRJNA263308.

## Electronic supplementary material

Additional file 1: **List of 362 conserved miRNA.** Transcript site counts, sequences and length for 362 conserved miRNA. (XLSX 24 KB)

Additional file 2: **List of novel miRNA.** Novel miRNAs identified in six libraries. For each library, A represents the novel miRNAs deposited in miRBase while B represents all the novel miRNAs identified in the study. MiRNA family, miRNA ID, location, length, GC%, MFE for precursor miRNA and count on 5p and 3p and total reads for mature miRNA are listed. (ZIP 483 KB)

Additional file 3: **Distribution of miRNAs on pear genome chromosomes.** The distribution of miRNAs in six libraries on each pear chromosome. (ZIP 147 KB)

Additional file 4: **List of miRNAs cluster.** 14 miRNA clusters at distances of 10 kb and 66 clusters at 100 kb. (ZIP 97 KB)

Additional file 5: **Differential expression profiles of conserved miRNAs in six libraries.** 15DAF is taken as control. The expression patterns of 362 conserved miRNA among the pear fruit development stages. With color closer to red, higher expression level is represented. (PNG 416 KB)

Additional file 6: **List of miRNA target genes.** A: Conserved and B: novel miRNA target genes. (ZIP 83 KB)

Additional file 7: **KEGG pathway annotation for all miRNA target genes.** A total of 235 KEGG pathways of the target genes annotated in the study. (ZIP 5 MB)
