# Supplementary figures and images for: Identification of miRNAs involved in pear fruit development and quality
Source: BMC Genomics. 2014 Nov 3;15(1):953. doi: 10.1186/1471-2164-15-953 (PMC4233070; doi:10.1186/1471-2164-15-953)

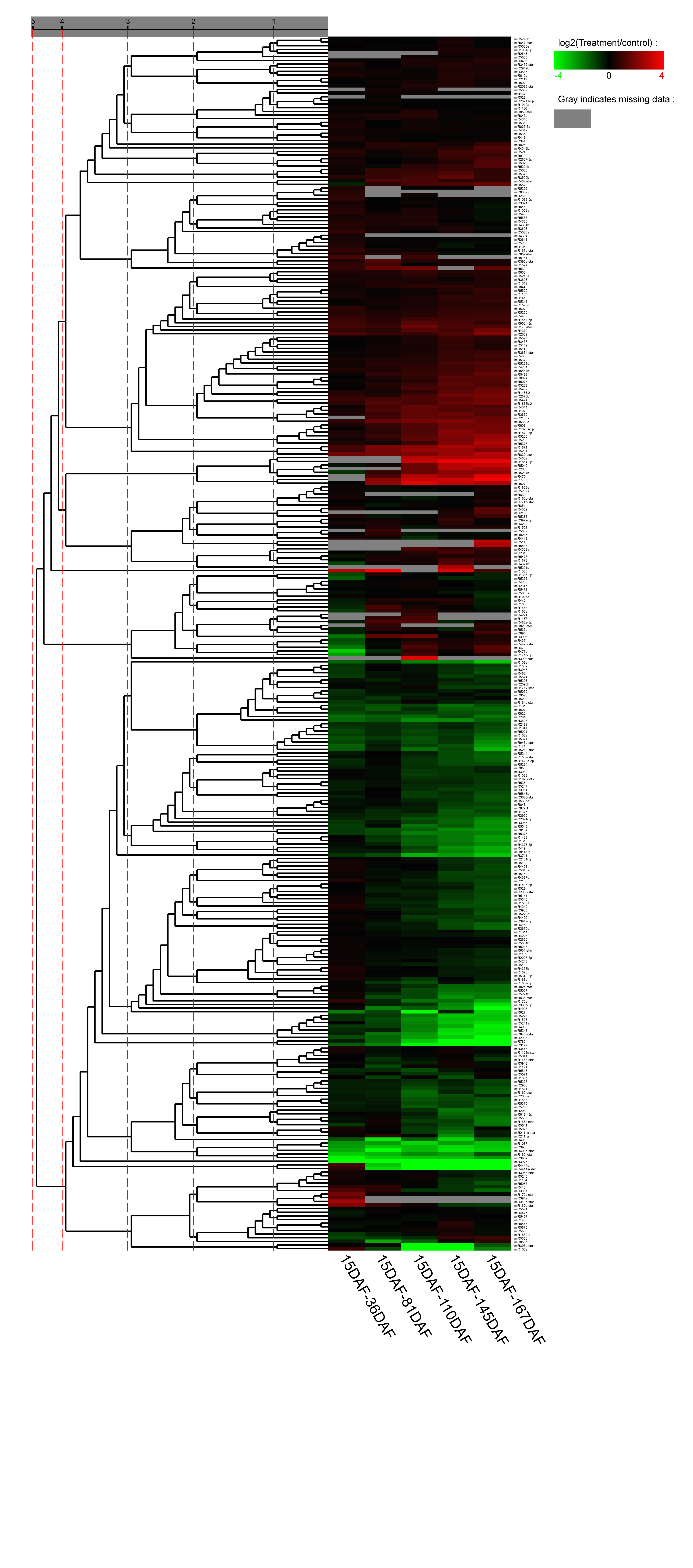

Supplement: Supplementary file 5 — Additional file 5: Differential expression profiles of conserved miRNAs in six libraries. 15DAF is taken as control. The expression patterns of 362 conserved miRNA among the pear fruit development stages. With color closer to red, higher expression level is represented. (PNG 416 KB) [file 12864_2014_6654_MOESM5_ESM.png]
